# Global Impact of COVID-19 on Weight and Weight-Related Behaviors in the Adult Population: A Scoping Review

**DOI:** 10.3390/ijerph18041876

**Published:** 2021-02-15

**Authors:** Han Shi Jocelyn Chew, Violeta Lopez

**Affiliations:** 1 Alice Lee Centre for Nursing Studies, Yong Loo Lin School of Medicine, National University of Singapore, Singapore 119077, Singapore; 2 School of Nursing, Hubei University of Medicine, 30 South Renmin Road, Shiyan 442000, China; hccasyd@gmail.com; 3 School of Nursing, University of Tasmania, Tasmania 7005, Australia

**Keywords:** COVID-19, weight, obesity, behavior, health, pandemic, adult, diet, physical activity, scoping

## Abstract

*Objective:* To provide an overview of what is known about the impact of COVID-19 on weight and weight-related behaviors. *Methods*: Systematic scoping review using the Arksey and O’Malley methodology. *Results*: A total of 19 out of 396 articles were included. All studies were conducted using online self-report surveys. The average age of respondents ranged from 19 to 47 years old, comprised of more females. Almost one-half and one-fifth of the respondents gained and lost weight during the COVID-19 pandemic, respectively. Among articles that examined weight, diet and physical activity changes concurrently, weight gain was reported alongside a 36.3% to 59.6% increase in total food consumption and a 67.4% to 61.4% decrease in physical activities. Weight gain predictors included female sex, middle-age, increased appetite, snacking after dinner, less physical exercise, sedentary behaviors of ≥6 h/day, low water consumption and less sleep at night. Included articles did not illustrate significant associations between alcohol consumption, screen time, education, place of living and employment status, although sedentary behaviors, including screen time, did increase significantly. *Conclusions:* Examining behavioral differences alone is insufficient in predicting weight status. Future research could examine differences in personality and coping mechanisms to design more personalized and effective weight management interventions.

## 1. Introduction

Since the COVID-19 pandemic emerged about a year ago, it has infected more than 72 million people and claimed above 1.5 million lives [1]. As of 8 December 2020, approximately 152 countries/territories have experienced some form of lockdown or confinement that curtailed social mobility to prevent the spread of the COVID-19. This includes changes in social norms such as working from home, hosting smaller social gatherings and reducing air travel. However, the impact of such measures on weight-related lifestyle behaviors and weight changes remains unclear. While some studies reported an increase in time for physical activities and preparing homemade food [2,3], others have reported an increase in sedentary behaviors [4], decreased physical activity [4], increased consumption of junk food and weight gain [5]. The COVID-19 pandemic is a novel disease of which its impact on the global adult obesity situation is unclear. More than 13% and 39% of the global adult population are obese and overweight, respectively. Current evidence highlights two worrying trends between COVID-19 and obesity, which could well form a vicious cycle: (1) COVID-19 associated with weight gain and (2) worse patient outcomes in patients with concurrent obesity and a COVID-19 infection [6,7,8].

Due to the novelty of this disease, the range, nature and magnitude of its impact on weight management in healthy adults remain unclear. Existing systematic reviews tend to focus on the outcomes of patients with obesity diagnosed with COVID-19, but the authors could not find systematic reviews on the effects of COVID-19 on weight and weight-related behaviors [9,10,11]. Therefore, a scoping review is timely and appropriate in mapping the current evidence on the impact of COVID-19 on weight management in healthy adults, specifically to identify literature gaps (not research gaps) to inform future research directions [12]. Although COVID-19 prevention measures such as reduced social mobility will gradually be weaned off with time, measures like working from home will most likely be a new norm. Therefore, conducting a scoping review would provide an overview of the current evidence on the impact of COVID-19 on weight management, identify research gaps and determine the need to conduct further systematic reviews to answer specific research questions [13]. The aim of this review was to investigate what is known about the changes in weight and weight-related behaviors in healthy adult populations during the COVID-19 pandemic.

## 2. Materials and Methods

This systematic scoping review was conducted according to the five-phased methodology developed by Arksey and O’Malley [14]. Scoping reviews are useful for exploring relatively new evidence and phenomenon that remains ambiguous in terms of what research questions to evaluate in a systematic review or primary research. Specifically, it is valued for identifying the breadth, key concepts and key conceptual factors of evidence available on a certain topic while identifying current knowledge gaps to guide the direction of future inquiries (e.g., conducting a systematic review). This differs from the objectives of conducting a systematic review that aims to analyze current evidence and answer specific research questions to guide decision-making, practice and policies [15]. The study findings are illustrated according to the preferred reporting items for systematic reviews and meta-analyses extension for scoping reviews (PRISMA-ScR) checklist (Table S1).

Phase 1: Research questions

This study’s research question was developed based on the population, intervention, comparison and outcome (PICO) framework to identify changes in weight and weight-related behaviors during the COVID-19 pandemic in healthy adult populations. Thus, the research question of this study was, “what is known about the changes in weight and weight-related behaviors in healthy adult populations during the COVID-19 pandemic?” Due to the limited number of studies that reports the impact of the COVID-19 pandemic on weight and weight-related behaviors, we included studies that examined populations with a majority of adults (i.e., mean age is >18) and excluded studies that reported exclusively on populations that were <18 years old. Studies on community-dwelling populations without diseases except being overweight or with obesity during this pandemic were included.

Phase 2: Literature search

A systematic three-step search strategy was used to identify relevant literature that was published up to 8 October 2020. First, search terms were generated iteratively through searches on CINAHL and PubMed using the keywords “weight”, “obesity,” and “COVID-19”. MeSH terms were also identified and used as search terms. Second, seven databases (CINAHL, Cochrane Central, Embase, PsycInfo, PubMed, Scopus and Web of Science) were searched for relevant articles published from the inception of the COVID-19 pandemic to 8 October 2020. The search terms used were “obes*”, “overweight”, “weight”, “COVID”, “COVID-19”, “SARS-COV2”, “SARS-CoV-2”, “2019-nCoV”, “2019 coronavirus”, “behavio*”. More information on the different combinations of the search terms used according to different databases is shown in Table S2. Lastly, the references of the included studies were searched for additional articles.

Phase 3: Study selection

Studies were included if they: (1) described the changes in weight or weight-related behaviors (e.g., dietary or physical activity) during the COVID-19 pandemic and (2) were on community-dwelling adults without mention of other diseases except for obesity and being overweight. Studies were excluded if they focused on: (1) biological changes due to a COVID-19 infection; (2) obesity as a risk factor of COVID-19 infections and outcomes; (3) did not discuss weight-related changes related to COVID-19; and (4) were non-primary studies, e.g., simulation/modeling studies.

A total of 396 articles were retrieved. After removing 144 duplicate articles, the remaining titles and abstracts were screened for eligibility, which 77 articles were eligible for full-text screening. After excluding articles with reasons shown in Table S3, 18 articles remained and were included in this scoping review.

Phase 4: Data charting

A data extraction form was created by HSJC and pilot tested on 5 studies. While doing so, common weight-related changes were identified, namely change in dietary behaviors, physical activity behaviors and other lifestyle behaviors. Therefore, the data extraction form was modified to expand the heading “weight-related changes” to the specific ones mentioned earlier. An excel spreadsheet was created to consolidate the extracted data according to the following headings—authors, year of publication, country of origin, study design, survey type, recruitment period, aim of study, follow-up, total number of participants, age, race, baseline BMI, BMI categories, BMI categories’ cutoff scores, proportion of participants overweight, weight change, weight measurement instruments, diet change, diet measurement instruments, physical activity change, physical activity instruments, other weight-related lifestyle behavior changes, predictors of weight, diet, physical exercise and other weight-related lifestyle behavior changes, the significance of change (statistically significant or not) and important results. Countries of origin were recoded into World Health Organization (WHO) regions, and articles were regrouped into those that evaluated changes in weight, diet and physical activities.

## 3. Results

Phase 5: Collating, summarizing and reporting the results

The 19 included articles represented 61,764 respondents, where the sample sizes of the articles ranged from 90–13,515 (median = 1844), mean/median age ranged from 19 to 47 years old with a median of 33.7 years old. 52.6% of the articles were from the European region (i.e., Belgium, Croatia, Italy, Poland, Spain, UK), 83.9% were cross-sectional descriptive studies, and all outcomes were collected through online self-report surveys (the usual method of data collection during the pandemic due to social distancing policy). The majority of the studies recruited participants during the months of April and May (72.2%) and comprised of more females than males (of the 17 studies that reported the proportion of female participants). Ten articles reported the participants’ mean baseline BMI that ranged from 20.7 kg/m^2^ to 27.7 kg/m^2^; nine reported the proportion of participants who were overweight at baseline (25–60%), and only five articles reported cutoff score used to classify one’s BMI as overweight (four studies used 25 kg/m^2^, only one used 23 kg/m^2^ from China). More information on the study characteristics is detailed in Table 1.

**Figure 1:**
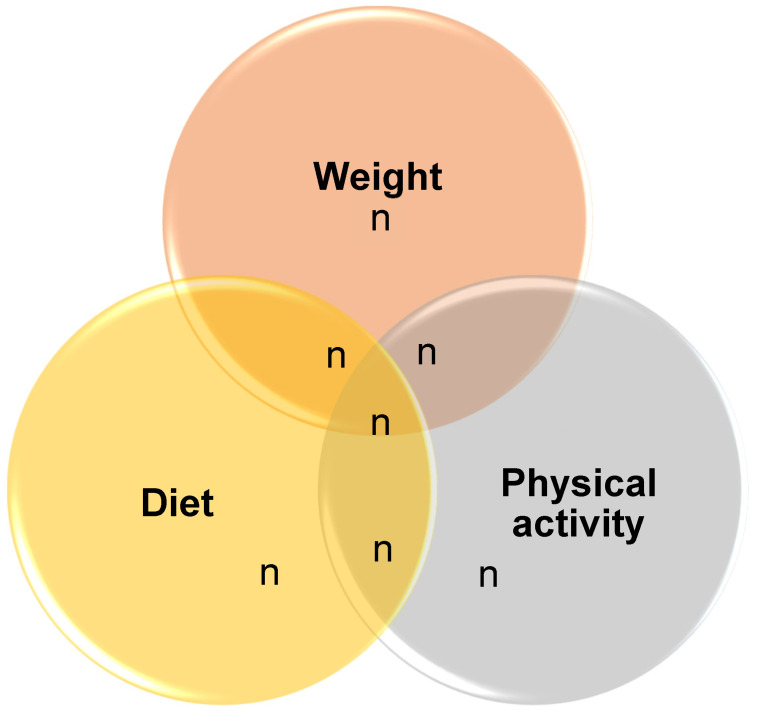
Number of articles on changes in weight, diet and physical activity.

Out of the 19 articles included in this scoping review [2,3,4,5,16,17,18,19,20,21,22,23,24,25,26,27,28,29,30], four explored changes in all three domains, namely weight, diet and exercise [2,4,5,24], three explored changes in both weight and diet and weight and exercise, three explored changes in both diet and exercise [22,23,24], and the rest explored each domain exclusively (Table 2, Figure 1). A summary of the impact of COVID-19 on the overall change in perceived weight status, dietary behaviors, physical activity behaviors, sedentary behaviors and other lifestyle behaviors are shown in Table 2.

### 3.1. Subsection

#### 3.1.1. Changes in Weight

Eleven out of 19 articles mentioned changes in weight where ten articles mentioned weight gain that ranged from 12.8% to 48.6% and six articles mentioned weight loss that ranged from 13.9–19.4% (Table 3) [2,3,4,5,18,19,21,25,26,28,29]. Two articles reported the combined proportion of participants who lost weight and did not perceive a change in weight [3,25]. It should be noted that these results were all derived from self-reports of perceived weight changes across different durations of confinement, cultural dietary norms (i.e., two studies focused on changes in Mediterranean diet change), and populations with different sociodemographic characteristics. Six studies examined the predictors of weight gain which included being in the middle-ages [4,26], female (n = 3; two studies reported odds ratio (OR) = 1.23–2.73) [2,4,18], higher baseline BMI (n = 3; two studies reported odds ratio OR = 1.07–1.12) [2,4,18], increased total food consumption [5], consumption of junk food (n = 2; OR = 1.76–3.12) [2,4], eating in response to sight and smell of food, stress eating and snacking after dinner [29], physical exercise (n = 5, three studies reported OR= 0.51–0.76), sedentary behavior ≥6 h/day (OR = 1.85), taking active breaks (OR = 0.72) [4], low water consumption (OR = 1.58) [4] and less hours of sleep a night [29]. However, one study did not find gender as a significant predictor of weight gain [26]. Alcohol consumption [18], screen time [5,29], education level [18,26], place of living and employment status [27] were also not significant predictors of weight gain. On the other hand, while not assessed for associations with weight changes, other lifestyle behavior changes were identified, including a general increase in sleep hours per night (30% to 54.8% of the respondents indicated an increase) [2,4,22,28,30], screen time per day (49.1% to 84.1% of the respondents indicated an increase) [5,22,28], stress/anxiety/boredom (42.7%) and concerns over weight, shape and eating [5]. However, there were contradictions regarding the changes in cigarette smoking per day [2,18].

#### 3.1.2. Changes in Dietary Behaviors

Eleven articles described changes in dietary behaviors in terms of appetite (34.4% increased, 17.8% decreased) [2], total consumption (34.3% to 59.6% increased, 5.7% to 33.5% decreased) [3,4,5,22,26], food type, adherence to a healthy diet (33.7% to 37% of the respondents increased, 26.7% to 32% decreased) [2,3,4,24,27], consumption of homemade meals (51.3% of the respondents increased, 14.9% decreased) [2], alcohol (generally decreased) [2,18], decreased coffee consumption especially in men [18], bingeing on food (49% of the respondents increased, 19% decreased) [24] and eating behaviors (59% of the respondents increased eating with friends and family, 65% of them increased eating in response to food stimuli, 73% increased eating due to food cravings, 52% increased stress eating, 73% increased bored eating and 65% increased snacking after dinner) (Table 4) [29]. Nine of the eleven studies reported changes in specific food and beverages, with two studies reporting specific Mediterranean diet using the Mediterranean diet adherence score (MEDAS) questionnaire [3,22]. Interestingly, one study found an initial increase in the proportion of participants consuming insufficient fruits and vegetables during the first week (52.8%; n = 58.1%) of confinement as compared to the pre-confinement period (49.3%) [23]. This consumption pattern increased steadily in participants experiencing confinement for the second and (48.8%; n = 22.4%) and third week (45.6%; n = 19.5%).

Being female was a significant predictor of increased appetite [2], increased consumption of homemade meals and healthy eating [3]. Age was a significant predictor of night snacking (OR = 0.97) [2], junk food consumption (OR = 0.98) [2], adherence to a Mediterranean diet (respondents aged 18–30 years had a higher MEDAS score compared to the younger and elder population) [2] and higher adherence to a healthy diet [3,22]. However, there were mixed findings regarding age as a predictor of dietary behavior. While one study reported a decrease in the likelihood of adopting a healthy diet with age (OR = 0.65, 0.33, 0.22 for 40 s, 50 s, more than 60 years old) [22], another study reported lower adherence to healthy diets in those aged 21 to 50 years old compared to those above 50 years old [3]. Only one study assessed the change in appetite that was shown to predict junk food consumption (OR = 4.04) and healthy eating (OR = 1.72) [2]. It was also associated with a change in work habits (e.g., working from home), BMI and being female. This study also did not find BMI and age as significant predictors of healthy eating. While one study reported that those from the North of Italy were less likely to have increased appetite (OR = 0.53) and have significantly higher adherence to a Mediterranean diet [2], another study reported that those from the North of Spain were less likely to adopt healthy eating habits (OR = 0.67) [3]. While one study reported that an increased BMI predicted an increase in appetite (OR = 1.07), junk food consumption (OR = 1.03) and lower adherence to a Mediterranean diet [2], another study reported that being overweight (OR = 1.31) or obese (OR: 1.64) were significant predictors of adherence to a healthy diet [22]. Adherence to an unhealthy diet was predicted by a decrease in physical activity (OR = 2.62), living in macroeconomic regions (OR = 1.43–1.47), increased screen time (OR = 1.54) and decreased consumption of homemade food (OR = 3.06) [5,22].

#### 3.1.3. Changes in Physical Activity and Sedentary Behaviors

Fourteen studies reported changes in physical activity, of which four studies reported on the changes in sedentary behaviors during the COVID-19 pandemic (Table 5). Physical activity was evaluated in terms of the overall level of activity and types of activities (i.e., walking, jogging, swimming, cycling, sports, weight lifting, and leisure-time activities). Four studies reported a higher proportion of respondents who increased rather than decreased physical activity (36–47%) [2,17,22,24] while seven studies reported the opposite in terms of proportion (35–70%) [3,4,5,16,21,28,30] mean duration (57.9 ± 34.5 to 51.1 ± 37.7 min/week (min/w)) [18] or metabolic equivalent task (median = 3006 to 1483.8 MET–min/w, median difference 1168.5 MET–min/w) [20]. Four studies reported a significant increase in sedentary behaviors, including increased sitting time (42.6% to 46%), TV watching, using electronics and social media [16,17,28,30]. One study reported an initial increase in the proportion of participants getting insufficient physical activity (<150 min/w) (35.1% to 52.2%) for participants experiencing confinement for the first week (n = 58.1%) but decreased in those participants experiencing confinement for the second and (40.3%; n = 22.4%) and third week (26.2%; n = 19.5%) [23].

Predictors of physical activity included being overweight (OR = 1.8), daily alcohol consumption (OR = 4.77), decreased vegetable consumption (OR = 3.32), perception of weight increase (OR-2.01), perception of having a healthy diet (OR = 2.11), eating more (OR = 1.87), sedentary for ≥6 h daily (OR = 2.12), exercise ≤30 min a day (OR = 1.99) [4] Other predictors were unhealthy eating [22], BMI, age, job type [16,20] and perceived time available [17]. There were mixed findings as to whether males decreased more significantly than females. [18,20] Instruments to estimate physical activity were mostly self-reports of, which only five studies used structured questionnaires, namely eating habits and lifestyle (EHLC)-COVID19 questionnaire [2], exercise comparison orientation measure [5], international physical activity questionnaire-short form (IPAQ-SF) [20,30], physical activity vital sign (PAVS) short version [23], IPAQ-Long Form [28] and sedentary behavior questionnaire (SBQ) [30].

#### 3.1.4. Changes in Other Lifestyle Behaviors

Nine studies examined changes in other lifestyle behaviors during the COVID-19 pandemic. Six studies reported an increase in sleep hours (49% to 49.9%) [2,4,22,23,28,30], while one reported an increase in smoking [16], two reported a decrease in smoking [18,23] and four studies reported an increase in screen time [5,22,23,28].

## 4. Discussion

Our scoping review found only 19 studies that examined COVID-19 related changes in weight and weight-related behaviors with mixed conclusions [2,3,4,5,16,17,18,19,20,21,22,23,24,25,26,27,28,29,30]. Moreover, all the studies were understandably conducted online due to the COVID-19 precautionary measures and derived from self-reports, which could have introduced risks of bias and inaccuracies in the study findings. Nevertheless, the study findings are valuable in addressing the obesity pandemic that could be worsened due to the COVID-19 pandemic and its induction of a new social norm-reduced social mobility.

From reviewing the included studies, up to approximately half of the respondents perceived weight gain during the COVID-19 pandemic period, while up to a fifth of the respondents had reportedly lost weight. There were also inconsistencies in the changes in dietary habits in terms of the consumption of healthy or junk foods and that of physical activity in terms of frequency, duration and energy expenditure. This could be associated with various individual characteristics and prepotent lifestyle habits that influenced weight-related lifestyle changes during the pandemic period. Respondents with a higher baseline BMI was shown to be more likely to experience weight gain, possibly due to a predisposition to eating in response to visual and olfactory food temptations, stress and emotional eating, as mentioned in the results section [2,3,4,5,18,22,26,29]. In general, the percentage increase (59.6%) in total consumption was more than that of a decrease (33.5%), and the adherence to a healthy diet increased slightly more than those who decreased [3,4,5,22,26]. This could explain the higher proportion of participants who gained weight despite having a higher adherence to a healthy diet due to a higher overall calorie consumed. However, further research is needed to support this speculation by using more objective calculations of energy intake and expenditure instead of using self-reported questionnaires that examine perceived intake change using Likert scales or “yes/no/no change” response options. Additionally, more than 50% of the respondents were reported to have increased eating episodes with friends and families in response to cravings, food stimuli and emotions [29]. Moreover, contrary to our speculation that COVID-19 decreases social eating, one study reported a 59% increase in social eating, specifically with family and friends [31]. This could be influenced by one’s personality traits and circumstances. For example, the frequency of social eating could have reduced during the COVID-19 pandemic, but while a more extroverted person may replace it with social eating with friends and families, one who is more introverted may not. In this case, the introverted individual could lose weight due to reduced total calorie consumption, but the extroverted individual could gain weight due to increased total calorie consumption. This is supported by a study that reported personality traits such as neuroticism, extraversion, agreeableness and conscientiousness to be significantly associated with health behaviors and self-efficacy in weight loss [32]. Future studies could consider exploring personality factors such as the Big five personalities when examining weight-related behavior trends to develop more personalized and targeted interventions.

Among the studies that examined changes in weight, diet and physical activity concurrently [2,4,5,24], weight gain was reported alongside an increase in total food intake in 36.3% to 59.6% of the respondents and a decrease in physical activity from 67.4% to 61.4% of the respondents. However, only one study examined and reported the association between increased eating and decreased physical activity [4]. Community-dwellers who were in the middle-ages and of the female sex were found to be more likely to gain weight, possibly due to an increased appetite, junk food consumption and total food consumption [2,4,18,26]. However, both predictors were also found to be associated with healthy eating, which suggests that weight gain could be associated with overconsumption (even for overconsumption of healthy food) or that these predictors only predicted a small change in weight status. Concurrently, one study reported that an increase in appetite predicted 1.7 to 4 times higher likelihood of junk food consumption and healthy eating, while another study reported a higher likelihood of healthy dietary adherence in individuals who were overweight [2,22]. Moreover, respondents, who were working from home, consumed less water, had less sleep at night, and stress eat could be more likely to gain weight. Other well-established predictors of weight gain were supported, including decreased physical activity, increased sedentary behavior and higher baseline BMI. However, there were mixed findings in terms of the proportion of respondents who increased versus decreased physical activity [2,3,4,5,16,17,21,22,24,28,30]. Therefore, while COVID-19 measures are to be in place for the next few years before they can be reasonably eradicated or be safe enough for the measures to be removed, health authorities could implement health promotion strategies to remind the citizens to be mindful of their total consumption (not only to eat more healthy foods) and stay physically active. This is especially for those who have a higher baseline BMI, of middle-age and of female sex as they are more likely to experience weight gain amidst a pandemic. Strategies could include teaching the public population on techniques to reduce appetite (e.g., taking small frequent meals), reduce snacking (e.g., distracting thoughts of snacking by performing physical activities), improve sleep (e.g., doing mindfulness exercises) and slotting physical exercises into their daily routines (e.g., taking the stairs instead of the lift).

Studies included in this review did not illustrate significant associations between weight gain and factors such as alcohol consumption, screen time, education, place of living and employment status, although sedentary behaviors and screen time did increase significantly [5,16,17,18,26,28,29,30]. This could suggest a moderating effect of screen time on the relationship between sedentary behaviors and weight gain, supported by a study where screen time seemed to be associated with weight gain only if it reduces physical activity, especially in adolescents [33]. An increase in screen time could also affect one’s sleep schedule and quality, an observed effect of the COVID-19 lockdown that is associated with weight change and depressive symptoms [34,35]. On the other hand, non-significant findings between socioeconomic status and weight gain contradict a study with a sample of 17,724 participants [33], possibly due to the relatively small sample sizes (N = 3027 and N = 1097) [18,26].

### Limitations

Our attempt at identifying the impact of COVID-19 on weight and weight-related behaviors was challenging because while some studies reported the statistical significance of the changes before and after the COVID-19 pandemic, others merely mentioned changes in proportion. Therefore, some changes could have been exaggerated or confounded by other variables such as seasonal changes in temperate countries that cause weight change. Moreover, the time period by which the changes occurred was unclear. It is possible that there exists a behavior change trajectory in coping with the pandemic, where such changes could normalize back to baseline once an individual gets used to the current circumstances—resulting in a minimal net weight change. However, such observation requires a longitudinal study design, which was only used in two studies that reported weight gain in 28.4% to 40% of the respondents [5,19]. Another limitation is in the self-reported nature of all the studies, where reported weight changes could be inaccurate due to different calibrations and types of weighing scales used. Furthermore, some studies estimated weight changes based on the participants’ perceived weight change by asking them if they gained, lost or maintained their weight. While we extracted potential predictors of weight and weight-related behavior changes, statistical conclusions could not be achieved because of the heterogeneity of data analysis methods used. While some reported the odds ratio and the statistical significance of each variable in a model tested, others only reported the proportion of respondents who expressed changes. Moreover, the included studies were not consistent in control variables, all of which could have given rise to the mixed findings on the aforementioned predictors. Lastly, we did not search for literature in other languages, such as Chinese literature, from Chinese databases as both authors were generally English-speaking. Searching for articles from Chinese databases could have provided a more geographically balanced overview of the topic of inquiry.

## 5. Conclusions

While existing studies suggested a higher proportion of people, who gained as compared to those who lost weight, findings regarding the predictors of diet and physical activity changes remain mixed. Moreover, none of the included studies examined other influencing factors of weight-related behaviors, such as personality factors, which could be strong determinants of weight change. Future research could focus on the predictors of different weight-related adaptations (i.e., increase or decrease in weight-related behaviors) and use more objective outcome measures to enhance the development and accuracy of predictive models for weight management interventions. Health promotion initiatives could also consider exploring the respondents’ needs and preferences in designing weight management programs instead of just prescribing recommendations to follow. Nonetheless, these findings highlighted two behavioral health adaptations—an increase and decrease in the adoption of a healthier lifestyle—to cope with the pandemic measures. This could inform further research, practice and policies in enhancing healthy coping behaviors in a post-COVID-19 era of new norms.

## Figures and Tables

**Table 1:** Study characteristics.

No.	Author, Year	Country	Study Design	Recruitment/Data Collection Period	Aim of Study	N	Population Characteristics	Age (Mean ± SD, Range * or Age Category)	Female (%)	BMI ^a^ (Mean ± SD) or BMI Category
1	Alomari 2020 [16]	Jordan	Cross-sectional descriptive	April–May 2020	To examine the effect of COVID-19-induced confinement on physical activity and sedentary behavior.	1844	General population of Jordanian adults aged >18 years	33.7 ± 11.3, 18–72	69.5	26.3 ± NS, 54.6, NS
2	Constandt 2020 [17]	Belgium	Cross-sectional descriptive	30 March–5 April 2020	To examine adults’ exercise levels and patterns during the COVID-19 lockdown.	13,515	General population of Flemish citizens	18–34 (27.1%) 35–54 (37.8%) 55–74 (35.1%)	50.5	NS, NS, NS
3	Di Renzo 2020 [2]	Italy	Cross-sectional descriptive	5–24 April 2020	To investigate the immediate impact of COVID-19 pandemic on eating habits and lifestyle changes.	3533	General population of Italian population aged ≥ 12 years.	40.03 ± 13.53, 12–86	76.1	27.66 ± 4.10, 32.5, NS
4	Đogaš 2020 [18]	Croatia	Cross-sectional descriptive	25 April–5 May 2020,	To investigate the effect of COVID-19 lockdown on lifestyle behaviors and mood changes.	3027	General population of Croatians aged >18 years	Median (IQR) = 4 0 (30–50)	79.7	24.64 ± 4.22, NS, NS
5	Ghosal 2020 [19]	India	Longitudinal	49 days pre and post confinement	To determine this risk of weight gain and type 2 diabetes mellitus (T2DM).	100	Non-diabetic household members of patients with T2DM.	<40 (59%) 40–49 (14%) 50–59 (18%) ≥60 (9%)	58	<25 kg/m^2^ (40%); 25–30 kg/m^2^ (42%); 30–<40 kg/m^2^ (18%), 60, 25
6	Giustino 2020 [20]	Italy	Cross-sectional descriptive	30 March–2 April 2020.	To estimate the levels of physical activity before and during the last seven days of the COVID-19 quarantine.	802	Physically active Sicilian population	32.27 ± 12.81	NS	23.44 ± 3.33, 25, NS
7	Gomes 2020 [21]	Brazil	Cross-sectional descriptive	29 April–10 May 2020	To evaluate the impact of COVID-19 on clinical practice, income, health and lifestyle behaviors.	766	Brazilian urologists	Median (IQR) = 46.0 (38–57)	NS	Median (IQR) = 26.5 (24.4–28.7), NS, NS
8	Górnicka 2020 [22]	Poland	Cross-sectional descriptive	30 April–23 May 2020	To identify dietary change patterns during the COVID-19 pandemic and their associations with sociodemographics, lifestyles and BMI before the pandemic.	2381	General population of Polish adults aged >18 years	<30 (39.4%) 30–39 (44.8%) 40–49 (12.9%) 50–59 (6.7%) ≥60 (6.1%)	89.8	< 18.5 kg/m^2^ (5.8%); 18.5–24.9 kg/m^2^ (58.2%); 25.0–30.0 kg/m^2^ (25.8%); ≥30.0 kg/m^2^ (10.2%), 36, 25
9	Keel 2020 [5]	US	Longitudinal	15–24 April 2020	To document perceived and observed longitudinal changes in reported weight, BMI, and how college students described their weight.	90	Undergraduates from a southeastern public university	19.45 ± 1.26	88	22.93 ± 4.02, NS, 25
10	López-Bueno 2020 [23]	Spain	Cross-sectional descriptive	22 March–5 April 2020	To investigate changes in health risk behaviors during the COVID-19 confinement.	2741	General population of Spain aged >18 years	34.2 ± 13.0	51.8	NS, NS, NS
11	Reyes-Olavarría 2020 [4]	Chile	Cross-sectional descriptive	May–June 2020	To determine lifestyle changes caused by COVID-19 confinement and analyze its association with changes in body weight.	700	General population of Chile aged >18 years	Median (range) = 31 (18–62)	82.6	Median (range) = 25.3 (20.2–34.95), 52.3, NS
12	Robinson 2020 [24]	UK	Cross-sectional descriptive	19–22 April 2020	To examine perceptions of how weight-related lifestyle changed in social lockdown with before the emergence of the COVID-19 crisis.	723	General population of UK aged >18 years	30.7 ± 9.6	67	< 18.5 kg/m^2^ (4%); 18.5–24.9 kg/m^2^ (51%); 25.0–30.0 kg/m^2^ (25%); ≥30.0 kg/m^2^ (20%), 45, 25
13	Rodríguez-Pérez 2020 [3]	Spain	Cross-sectional descriptive	20 March–3 April 2020	To evaluate dietary behavior changes during the COVID-19 outbreak confinement.	7514	General population of Spain aged >18 years	<20 (3.0%) 21–35 (34.0%) 36–50 (31.6%) 51–65 (25.7%) >65 (5.7%)	70.6	NS, NS, NS
14	Scarmozzino 2020 [25]	Italy	Cross-sectional descriptive	3–15 April 2020	To assess the effects of COVID-19-induced confinement policies on self-reported food consumption.	1929	General population of Italy	<20 (14.4%) 21–35 (63.1%) 36–50 (9.6%) 51–65 (11.4%) >65 (1.5%)	67	NS, NS, NS
15	Sidor 2020 [26]	Poland	Cross-sectional descriptive	17 April–1 May	assess whether nutritional and consumer habits have been affected under these conditions.	1097	General population of Poland aged >18 years	27.7 ± 9.0 (18–71)	95.1	23.5 ± 4.8 (range = 14.4–57.8), 28.4, NS
16	Steele 2020 [27]	Brazil	Cohort	1^st^: 26 January–15 February 2020, 2^nd^ May 10–19, 2020	To describe the dietary characteristics of participants immediately before and during the COVID-19 pandemic.	10,116	Adults from the NutriNet Brazil cohort	18–39 (51.1%) 40–59 (39.9%) ≥60 (9.0%)	78	NS, NS, NS
17	Yang 2020 [28]	China	Cross-sectional descriptive	Early May 2020	To assess changes in obesity and activity patterns during COVID-19 lockdown.	10,082	General population of China	19.8 ± 2.3	71.7	21.8 ± 5.7, 31.8, 23
18	Zachary 2020 [29]	US	Cross-sectional descriptive	NS	To quantify the impact that self-quarantine has on behaviors associated with weight gain.	173	General population of US aged >18 years	28.1 ± 12.5	55.5	NS, NS, NS
19	Zheng 2020 [30]	Hong Kong	Cross-sectional descriptive	15–26 April 2020	To investigate: (1) physical activity levels and sleep during the COVID-19 epidemic, (2) change in these behaviors before and during the pandemic.	631	Young adults aged between 18 and 35	21.1 ± 2.9 (18–35)	61.2	20.7 ± 2.6, NS, NS

*Note:* SD = standard deviation; * = when mentioned; BMI = body mass index; NS = not specified; IQR = interquartile range; ^a^ baseline BMI; percentage change in BMI, cutoff BMI for being overweight

**Table 2:** Overall change in perceived weight status, dietary behaviors, physical activity behaviors, sedentary behaviors and other lifestyle behaviors.

No.	Author, Year	Perceived Weight Changes	Dietary Behavior Changes	Physical Activity Changes	Sedentary Behavior Changes	Other Lifestyle Behaviors Changes
1	Alomari 2020 [16]	NS	NS	Walking: 42.2% decreased, 33.8% increased Jogging: 41.8% decreased, 21.0% increased Swimming: 44.5% decreased, 6.5% increased Cycling: 26.6% decreased, 20.3% increased Sports: 41.6% decreased, 18.9% increased Weight lifting: 35.9% decreased, 17.9% increased	TV watching time: 5.6% decreased, 72.3% increased Using electronics: 3.2% decreased, 82.7% increased Logging to social media: 3.0% decreased, 81.9% increased	NS
2	Constandt 2020 [17]	NS	NS	36% increased, 23% decreased	Sitting time: 46% sits more, 15% sits less	NS
3	Di Renzo 2020 [2]	48.6% gained weight 13.9% lost weight	Appetite: 34.4% increased, 17.8% decreased Food type: increase in homemade recipes (e.g., sweets, pizza and bread), cereal, legumes, white meat, hot beverage. Decrease in fresh fish, packaged and baked products and delivery of food. Alcohol: decreased Junk food (packaged sweets, baked products, sweet beverages, savory snacks, dressing): more people decreased (29.8%) than increased Healthy eating (MEDAS): 37% increased	Increased training frequency among those already highly active, especially bodyweight training (38.3% of respondents) Those who train five or more days a week increased from 6 to 16%	NS	Sleep hours increased: 7–9 h/night (49.9% to 54.8%); >9 h/night (1.4% to 9.1%) Smoking decreased: no (74.9% to 78.2%); <5 cigarettes/day (8.9% to 8.2%); 5–10 cigarettes/day (8.3% to 6.3%); >10 cigarettes/day (7.9% to 7.3%)
4	Đogaš 2020 [18]	30.7% gained weight	NS	Decreased (mins): 57.9 ± 34.5 to 51.1 ± 37.7	NS	Smoking increased: 12.3 ± 7.8 to 14.3 ± 10.3 cigarettes/day)
5	Ghosal 2020 [19]	40% gained up to 5 kg of weight	NS	NS	NS	NS
6	Giustino 2020 [20]	NS	NS	Decreased total energy expenditure: 3006 to 1483.8 MET–min/w	NS	NS
7	Gomes 2020 [21]	32.9% gained weight 19.4% lost weight	NS	60% decreased	NS	NS
8	Górnicka 2020 [22]	NS	Total intake: 34.3% increased, 14.1% decreased	43% increased	NS	Screen time: 49.1% increased, 5.1% decreased Sleep time: 30% increased, 9.3% decreased
9	Keel 2020 [5]	28.4% gained weight 15.9% lost weight (no significant change in actual self-reported weight)	Total intake: 55.7% increased	61.4% decreased, 24.9% increased	NS	Watching TV/movies: 75% increased Time on social media (Instagram, Snapchat, Facebook): 84.1% increase Time on gaming: 29.6%, 58.9% no change Concerns about weight and shape: 65.9% increased Concerns about eating: 60.9% increase
10	López-Bueno 2020 [23]	NS	Insufficient fruits and vegetables consumption: increased from 49.3% to 52.8% for participants experiencing confinement first week (n = 58.1%), but decreased in those participants experiencing confinement for the second and (48.8%; n = 22.4%) and third week (45.6%; n = 19.5%) Alcohol consumption: decreased consistently from 70.5% to 53.4%, 46.5% and 43.3% in those experiencing confinement for the first, second and third week.	Insufficient physical activity (<150 min/week): increased from 35.1% to 52.2% for participants experiencing confinement for the first week (n = 58.1%), but decreased in those participants experiencing confinement for the second and (40.3%; n = 22.4%) and third week (26.2%; n = 19.5%)	NS	<6 h of sleep a day: decreased from 6.3% to 5% for participants experiencing confinement for the first week to 2.4% in those participants experiencing confinement for the second week and increased to 3.7% and for those in the third week. >2 h of screen time a day: increased from 83% to 97.7% in those experiencing confinement for the first week to 96.9% and 98.7% in those experiencing confinement for the second and third week.
11	Reyes-Olavarría 2020 [4]	35% gained weight 15.7% lost weight	Total intake: 59.6% increased, 5.7% decreased Homemade meals: 51.3% increased, 14.9% decreased Healthy eating: 33.7% increased, 26.7% decreased	57.4% decreased	NS	Sleep: 49% increased, 23% decreased
12	Robinson 2020 [24]	NS	Healthy eating: 30% increased, 32% decreased Bingeing on food: 49% increased, 19% decreased	35% decreased, 47% increased	NS	NS
13	Rodríguez-Pérez 2020 [3]	12.8% gained weight 47.3% did not (lost/no change)	Total intake: 36.3% increased Healthy eating (MEDAS): increased significantly from 6.53 ± 2 to 7.34 ± 1.93	59.6% decreased, 15.9% increased	NS	NS
14	Scarmozzino 2020 [25]	19.5% gained weight 50.7% did not (lost/no change)	Total intake: 52.9% increased, 33.5% decreased	NS	NS	42.7% said weight gain due to stress/anxiety bored 1.3% said weight gain due to increased price 49.6% did not change
15	Sidor 2020 [26]	29.9% gained weight 18.6% lost weight	Total intake: 43.5% increased	NS	NS	NS
16	Steele 2020 [27]	NS	Eating healthily: Increased significantly Eating unhealthily: increased, but not significant	NS	NS	NS
17	Yang 2020 [28]	BMI 21.8–22.6, *p* < 0.001 21.3–25.1%, increase in the prevalence of overweight/obesity	NS	Significant decreases in the frequency of commuting/errands (*p* < 0.001), leisure-time MVPA (*p* < 0.05), and leisure-time walking (*p* < 0.001).	During workdays: 42.7% increased, 21.3% decreased During weekends: 42.6% increased, 20% decreased	Sleep time (workdays): 35% increased, 19.6% decreased Sleep time (wee ends): 29% increased, 20.5% decreased Screen time: 36% increased, 7% decreased
18	Zachary 2020 [29]	22% gained 5–10 lbs 15% lost 5–10 lbs	Eat with friends and family: 59% increased Eat in response to sight and smell of food: 65% increased Eat because of food cravings: 73% increased Stress eating: 52% increased Bored eating: 73% increased Snacking after dinner: 65% increased	NS	NS	NS
19	Zheng 2020 [30]	NS	NS	70% decreased in physical activity	Increased from 7.8 ± 3.2 to 10.0 ± 3.2	Sleep time: increased 7.7 ± 1.0 to 8.4 ± 1.2 h/night

*Note:* NS = not specified; MEDAS= Mediterranean diet adherence screener; BMI = body mass index; MVPA = moderate to vigorous physical activity.

**Table 3:** Changes in weight and the corresponding predictors during the COVID-19 pandemic.

Authors	Weight Change	Predictors of Weight Change	Non-Significant Predictors
Di Renzo 2020	48.6% gained weight 13.9% lost weight	Consumption of junk food (OR = 3.122) Consumption of healthy food (OR = 0.805) BMI (OR = 1.073) Female (OR = 1.234) PA (OR = 0.66) From North Italy (OR = 0.786) From Central Italy (OR = 0.747)	NS
Đogaš 2020	30.7% gained weight	Female sex (OR = 2.726) BMI (OR = 1.116) PA (OR = 0.756)	Alcohol consumption Education level
Ghosal 2020	40% gained up to 5 kg of weight	NS	NS
Gomes 2020	32.9% gained weight 19.4% lost weight	NS	NS
Keel 2020	28.4% gained weight 15.9% lost weight (However, **no significant** change in actual self-reported weight)	Increased eating PA Higher weight/shape concerns Higher eating concerns	Watching TV/movies Social media use Gaming
Reyes-Olavarría 2020	35% gained weight 15.7% lost weight	Adjusted for age and sex (sig diff): Separated marital status (OR = 3.33) Married (OR = 1.52) Middle SES (OR = 1.48) Consumption of fried foods ≥3 times per week (OR = 3.36) Consumption of junk food ≥3 times per week (OR = 1.76) Low water consumption (OR = 1.58) Low consumption of legumes once per week (OR = 2.27) Low consumption of fish (OR = 0.67) PA ≥4 times per week (OR = 0.51) Active breaks (OR = 0.72) Sedentary behavior ≥6 h/day (OR = 1.85)	
Rodríguez-Pérez 2020	12.8% gained weight 47.3% did not (either lost or no change)	NS	NS
Scarmozzino 2020	19.5% gained weight 50.7% did not (either lost or no change)	NS	NS
Sidor 2020	29.9% gained weight 18.6% lost weight	BMI (particularly in overweight and obese subjects) Age (35–45 and >45 years old)	Education level Place of living Occupation status Gender
Yang 2020	BMI 21.8–22.6, increase in the prevalence of overweight/obesity	NS	NS
Zachary 2020	22% gained 5–10 lbs 15% lost 5–10 lbs	Eat in response to sight and smell of food Stress eating Snacking after dinner Hours of sleep per night Physical activity per week	Screen time

*Note:* NS = not specified; BMI = body mass index.

**Table 4:** Changes in dietary behaviors and the corresponding predictors during the COVID-19 pandemic.

Authors	Change in Dietary Behaviors	Predictors of Dietary Behaviors Change	Non-Significant Predictors
Di Renzo 2020	Appetite: 34.4% increased, 17.8% decreased Food type: Increase in homemade recipes, cereal, legumes, white meat, hot beverage. Decrease in fresh fish, packaged and backed products, delivery food. Alcohol: decreased Junk food: more people decreased (29.8%) than increased junk food consumption Healthy eating: 37% increased	Appetite: Change in work habits Female BMI (OR = 1.073) North and Central Italy compared to the South and Islands (OR = 0.527, OR = 0.582,). (post hoc test showed that younger increased appetite) Reduced appetite r/t healthy food intake Night snack: Age (OR = 0.972) Living in Central and Southern Italy (OR = 1.843) Junk Food: BMI (OR = 1.025) Age (OR = 0.979) Appetite (OR = 4.044) Healthy eating: Reduced appetite (OR = 1.718) Adherence to the Mediterranean diet: Significant higher in Northern and Southern Italy and Islands compared to Central Italy Inverse correlation between MEDAS score, BMI and age 18–30 years old adults had a higher MEDAS score compared to the younger and the elder population	Healthy eating: BMI and age
Dogas 2020	Alcohol: decreased for those who never drinks (19.1–32.1%), once monthly (31.9% to 22.3%), up to 3 drinks weekly (32.3–27.2%), but increased for those who drinks more than 7 drinks weekly Coffee per day: men decreased 2.4 ± 1.2 to 2.0 ± 1.2	NS	NS
Górnicka (2020)	Total intake: 34.3% increased, 14.1% decreased	Adherence to a healthy diet: Age (OR = 0.65, 0.33, 0.22 for 40 s, 50 s, more than 60 y) Being overweight (OR = 1.31) or obese (OR = 1.64) before the pandemic Increased physical activity (OR = 1.53) Increased consumption of homemade food (OR = 2.32) Adherence to unhealthy diet: Living in macroeconomic regions (OR = 1.43–1.47) Decreased physical activity (OR = 2.62) Increased screen time (OR = 1.54) Decreased consumption of homemade food (OR = 3.06)	NS
Keel 2020	Total intake: 55.7% increased	Total intake: Watching television	NS
López-Bueno 2020	Insufficient fruits and vegetables consumption: increased from 49.3% to 52.8% for participants experiencing confinement first week (n = 58.1%), but decreased in those participants experiencing confinement for the second and (48.8%; n = 22.4%) and third week (45.6%; n = 19.5%) Alcohol consumption: decreased consistently from 70.5% to 53.4%, 46.5% and 43.3% in those experiencing confinement for the first, second and third week.	NS	NS
Reyes-Olavarría 2020	Total intake: 59.6% increased, 5.7% decreased Homemade meals: 51.3% increased, 14.9% decreased Healthy eating: 33.7% increased, 26.7% decreased	Homemade meals: Female	NS
Robinson 2020	Healthy eating: 30% increased, 32% decreased Bingeing on food: 49% increased, 19% decreased	NS	NS
Rodríguez-Pérez 2020	Total intake: 36.3% increased Healthy eating: increased significantly from a mean score of 6.53 ± 2 to 7.34 ± 1.93	Healthy eating: Age (>50 y OR = 0.9) (21 to 50 years old lower adherence than >50 y) Female Higher educational level (postgrad OR = 1.13) Region (north of Spain compared to other regions OR = 0.67) Lived alone (OR = 1.36) Never performed physical activity (OR = 0.78)	NS
Scarmozzino 2020	Total intake: 52.9% increased, 33.5% decreased	42.7% attribute it to anxiety	
Sidor 2020	Total intake: 43.5% increased	NS	NS
Steele 2020	Eating healthily: Increased significantly, ref to specific diet sheet Eating unhealthily: increased, but not sig	NS	NS
Zachary 2020	Eat with friends and family: 59% increased Eat in response to sight and smell of food: 65% increased Eat because of food cravings: 73% increased Stress eating: 52% increased Bored eating: 73% increased Snacking after dinner: 65% increased	NS	NS

*Note:* NS = not specified; BMI = body mass index; MEDAS = Mediterranean diet adherence screener.

**Table 5:** Changes in physical activity behaviors and the corresponding predictors during the COVID-19 pandemic.

Authors	Measurement Instrument	Significant Changes in Physical Activity Factors	Predictors of Physical Activity Change	Significant Changes in Sedentary Behaviors Factors	Predictors of Sedentary Behaviors Change
Alomari 2020	Self-report questions	Walking: 42.2% decreased, 33.8% increased Jogging: 41.8% decreased, 21.0% increased Swimming: 44.5% decreased, 6.5% increased Cycling: 26.6% decreased, 20.3% increased Sports: 41.6% decreased, 18.9% increased Weight lifting: 35.9% decreased, 17.9% increased	Walking: gender, job type (those in the military, agriculture, health and engineering less likely to express increase than no change) Jogging: age, job type Cycling: age Weightlifting: age and obesity Swimming: age and obesity Sports: age, gender	TV watching time: 5.6% decreased, 72.3% increased Using electronics: 3.2% decreased, 82.7% increased Logging to social media: 3.0% decreased, 81.9% increased	TV watching time: age, gender, obesity, income Using electronics: education, income, job type Logging to social media: 3.0% decreased, 81.9% increased
Constandt 2020	Self-report questions	36% increased, 23% decreased	Having less time, sitting more, and missing the familiar way and competitive element of exercising reduced exercise Perceived time available: 54% perceived more time to exercise, 6% less time Previously low active adults exercised more during the lockdown except for people aged > 55	Sitting time: 46% sits more, 15% sits less	Closed sports infrastructure (50%) Non-presence of sport club activities (38%) Canceled sports events (32%) Absence of friends to exercise with (30%)
Di Renzo 2020	EHLC-COVID19 questionnaire	Higher frequency of training among those who were already highly active Those who train five or more days a week increased from 6 to 16% A slight increase in physical activity, especially for bodyweight training (38.3% of respondents).	Possibly more time	NS	NS
Đogaš 2020	Self-report questions	Decreased (mins): 57.9 ± 34.5 to 51.1 ± 37.7	Women decreased exercise significantly in terms of duration (55.6 ± 29.8 to 49.2 ± 32.5) and frequency (2.8 ± 1.2 to ± 2.7 to 1.2) No sig changes in exercise for men	NS	NS
Giustino 2020	IPAQ-SF	Decreased total weekly energy expenditure: 3006 to 1483.8 MET–min/week	BMI Age Males decreased more than females	NS	NS
Gomes 2020	Self-report questions	60% deduced	Reduction in gym/personal trainer (45.1%) Reduced supermarket run (34.5%)	NS	NS
Górnicka	Self-report questions	43% increased	65% of respondents in the unhealthy pattern had reduced PA.	NS	NS
Keel 2020	Exercise comparison orientation measure	61.4% decreased, 24.9% increased	NS	NS	NS
López-Bueno 2020 [23]	Physical activity vital sign (PAVS) short version	Insufficient physical activity (<150 min/week): increased from 35.1% to 52.2% for participants experiencing confinement for the first week (n = 58.1%), but decreased in those participants experiencing confinement for the second and (40.3%; n = 22.4%) and third week (26.2%; n = 19.5%)	NS	NS	NS
Reyes-Olavarría 2020	Self-report questions	57.4% decreased	Perception of weight increase (OR = 2.01) Being overweight (OR = 1.8) Daily alcohol consumption (OR = 4.77) Decreased vegetable consumption (OR = 3.32) Perception of having a healthier diet (OR = 2.11) Eating more food than before (OR = 1.87) Sedentary ≥6 h (OR = 2.12) Exercise session duration ≤30 min (OR = 1.99) Yoga and pilates (OR = 1.82) Physical activity 1–3 times per week (OR = 1.67)	NS	NS
Robinson 2020	Self-report questions	35% decreased, 47% increased	NS	NS	NS
Rodríguez-Pérez 2020	Self-report questions	59.6% decreased, 15.9% increased	NS	NS	NS
Yang 2020	IPAQ-LF	Significant decreases in the frequency of engaging in active transport for commuting/errands (*p* < 0.001), leisure-time MVPA (*p* < 0.05), and leisure-time walking (*p* < 0.001).	NS	During workdays: 42.7% increased, 21.3% decreased During weekends: 42.6% increased, 20% decreased	NS
Zheng 2020	IPAQ-SF, sedentary behavior questionnaire (SBQ)	70% decreased in PA, including VPA, MPA and walking.	NS	Increased from 7.8 ± 3.2 to 10.0 ± 3.2	Increased engagement in TV/DVD (0.9 ± 0.8 vs. 1.7 ± 1.4) Increased computer/paper work (2.2 ± 1.7 to 3.1 ± 2.0) Decreased sitting time during transportation (0.7 ± 0.7 vs. 0.4 ± 0.6)

*Note:* NS = not specified; EHLC-COVID19 = eating habits and lifestyle changes in COVID-19 lockdown; IPAQ-SF = international physical activity questionnaire-short-form; IPAQ-LF = International physical activity questionnaire long-form.

## Data Availability

Data is contained within the article or Supplementary Material.

## Supplementary Materials

The following are available online at https://www.mdpi.com/1660-4601/18/4/1876/s1, Table S1: preferred reporting items for systematic reviews and meta-analyses extension for scoping reviews (PRISMA-ScR) checklist; Table S2: Search terms used in each corresponding electronic database; and Figure S3: PRISMA flow diagram.
